# Neuronal Activity Induces Synaptic Delivery of hnRNP A2/B1 by a BDNF-Dependent Mechanism in Cultured Hippocampal Neurons

**DOI:** 10.1371/journal.pone.0108175

**Published:** 2014-10-06

**Authors:** Graciano Leal, Pedro M. Afonso, Carlos B. Duarte

**Affiliations:** 1 CNC-Center for Neuroscience and Cell Biology, University of Coimbra, Coimbra, Portugal; 2 Department of Life Sciences, University of Coimbra, Coimbra, Portugal; 3 Faculty of Pharmacy, University of Coimbra, Coimbra, Portugal; Institut National de la Santé et de la Recherche Médicale (INSERM U901), France

## Abstract

Dendritic protein synthesis plays a critical role in several forms of synaptic plasticity, including BDNF (brain-derived neurotrophic factor)-mediated long-term synaptic potentiation (LTP). Dendritic transcripts are typically transported in a repressed state as components of large ribonucleoprotein complexes, and then translated upon stimulation at, or in the vicinity, of activated synapses. Heterogeneous nuclear ribonucleoprotein A2/B1 (hnRNP A2/B1) is a *trans-*acting factor involved in dendritic mRNA trafficking, but how the distribution of the protein in dendrites is regulated has not been characterized. Here we found that a fraction of hnRNP A2/B1 is present at the synapse under resting conditions in cultured hippocampal neurons. Accordingly, this ribonucleoprotein was detected in free mRNP, monosomal, and polyribosomal fractions obtained from synaptoneurosomes. Neuronal activity and BDNF treatment increased hnRNP A2/B1 protein levels in the cell body and dendritic compartments, and induced the delivery of this protein to synaptic sites. The activity-dependent accumulation of hnRNP A2/B1 at the synapse required, at least in part, the activation of TrkB receptors, presumably by BDNF. This neurotrophin also upregulated the hnRNP A2/B1 mRNA in the soma but was without effect on the abundance of neuritic hnRNP A2/B1 transcripts. These results show that the distribution of hnRNP A2/B1 is regulated by BDNF and by neuronal activity, an effect that may have a role in BDNF-induced synaptic plasticity events.

## Introduction

Experience-dependent changes in synapse structure and function are thought to underlie learning and memory formation [1]. Some of these modifications require activity-dependent transport and translation of dendritic-localized mRNAs, with concomitant local alterations in the proteome [2]. These biochemical alterations, together with structural and functional modifications, are required for several forms of synaptic plasticity, including BDNF-mediated LTP [3].

Dendritic mRNAs are usually packaged into large messenger ribonucleoprotein complexes (mRNPs) in the cell body and transported along the microtubule cytoskeleton until they reach their destination. During this process, the transcripts are usually kept in a dormant state and then translated upon synaptic activation [4]. One of the best described RNA-binding proteins involved in mRNA trafficking is the hnRNP A2/B1, which recognizes a *cis*-acting element present in the myelin basic protein (MBP) mRNA [5], [6]. Because it is recognized by hnRNP A2/B1, that element is known as hnRNP A2 response element (A2RE). In neurons, the A2RE-dependent targeting of mRNAs is involved in the dendritic delivery of activity-regulated cytoskeleton-associated protein (Arc), CaMKIIα, and Neurogranin mRNAs, which appear to cluster in the same hnRNP A2/B1-containing granules [7]. Moreover, hnRNP A2/B1 is necessary for the delivery of the noncoding BC1 RNA and PKMζ mRNA to distal dendritic domains [8], [9]. Given the nature of some of the hnRNP A2/B1-associated transcripts, the protein may play a role in long-term synaptic potentiation.

Several transcripts are transported to dendrites upon synaptic activity, including mRNAs that contain A2RE-like elements in their sequences (e.g. Arc, CaMKIIα, BDNF) [7], [10]–[18], suggesting that the transport of hnRNP A2/B1 in dendrites may be regulated by neuronal activity. Here we show that hnRNP A2/B1 protein exhibits a punctate distribution in dendrites of hippocampal neurons and is in part present at the synapse under basal conditions. Moreover, hnRNP A2/B1 was identified in monosomal- and polyribosomal-associated fractions obtained from rat hippocampal synaptoneurosomes, a subcellular fraction containing the pre- and postsynaptic regions. We also found that synaptic activity and the neurotrophin BDNF increase the levels of cell body- and dendritic-localized hnRNP A2/B1 protein and induce its accumulation in synaptic sites. Importantly, BDNF mediates the synaptic accumulation of hnRNP A2/B1 induced by neuronal activity.

## Materials and Methods

### Ethics Statement

Experiments were performed according to the European Union Directive 86/609/EEC and the legislation Portaria n. 1005/92, issued by the Portuguese Government for the protection of animals used for experimental and other scientific purposes. Dams were sacrificed by cervical dislocation. Embryos were then surgically removed and sacrificed by decapitation.

### Hippocampal cultures

Low-density hippocampal cultures were prepared as previously described [19], [20]. Briefly, hippocampi were dissected from E18 rat embryos and the cells were dissociated using trypsin (0.25%). Neurons were plated at a final density of 1–5×10^4^ cells/dish on poly-D-lysine-coated glass coverslips in neuronal plating medium (MEM supplemented with 10% horse serum, 0.6% glucose and 1 mM pyruvic acid). After 2–4 h, coverslips were flipped over an astroglial feeder layer in Neurobasal medium (GIBCO - Life Technologies) supplemented with SM1 supplement (1∶50 dilution, STEMCELL Technologies), 25 µM glutamate, 0.5 mM glutamine and 0.12 mg/ml gentamycin (GIBCO - Life Technologies). The neurons grew face down over the feeder layer but were kept separate from the glia by wax dots on the neuronal side of the coverslips. To prevent overgrowth of glial cells, neuron cultures were treated with 5 µM cytosine arabinoside (Sigma-Aldrich) after 3 DIV. Cultures were maintained in a humidified incubator with 5% CO_2_/95% air, at 37°C, for up to 2 weeks, feeding the cells once per week. At DIV 14–15 neurons were stimulated for 30 min with 100 ng/ml BDNF (Peprotech) or with 50 µM bicuculline (Tocris), 2.5 mM 4-aminopyridine (4-AP) (Tocris) and 10 µM glycine (Sigma-Aldrich) to increase synaptic activity. Where indicated, cells were pre-treated for 30 min with the Trk receptor inhibitor SHN722 (1 µM) [21], [22] or with the scavenger of extracellular TrkB receptor ligands TrkB-Fc (1 µg/ml) (R&D Systems) before stimulation with 100 ng/ml BDNF (Peprotech) or with the cocktail solution containing bicuculline (50 µM bicuculline, 2.5 mM 4-AP and 10 µM glycine), respectively.

High-density hippocampal cultures were prepared from the hippocampi of E18-E19 Wistar rat embryos as described previously [23] and the cells plated (80 000 cells/cm^2^) in 3 µm pore 24 mm polyethylene terephthalate (PET) membrane filter inserts (Corning) coated with poly-D-lysine (0.1 mg/ml) [24], [25]. The cultures were maintained in a humidified incubator of 5% CO_2_/95% air, at 37°C, for 14–15 days and then stimulated with 100 ng/ml BDNF (Peprotech) for the indicated periods of time.

### Immunocytochemistry

Hippocampal neurons (low density) were fixed in 4% sucrose/paraformaldehyde (in PBS) for 15 min at room temperature and permeabilized with 0.3% Triton X-100 in PBS. The neurons were then incubated with 10% BSA in PBS for 30 min at 37°C to block non-specific staining, and incubated overnight at 4°C with the primary antibodies diluted in 3% BSA in PBS. The following primary antibodies and dilutions were used: anti-hnRNP A2/B1 (sc-53531, 1∶200; Santa Cruz Biotechnology), anti-MAP2 (ab5392, 1∶10.000; Abcam), anti-PSD95 (D27E11, 1∶200; Cell Signaling). The cells were washed 6 times with PBS for 2 min and incubated with Alexa Fluor 568 (1∶500, Invitrogen), Alexa Fluor 488 (1∶500; Invitrogen) and AMCA (1∶200; Jackson ImmunoResearch) conjugated secondary antibodies, for 45 min at 37°C. After washing the cells 6 times with PBS for 2 min, the coverslips were mounted with a fluorescence mounting medium (DAKO).

### Microscopy and quantitative fluorescence analysis

Imaging was performed on a Zeiss Observer Z.1 microscope using a 63×1.4 NA oil objective. Images were quantified using the ImageJ image analysis software as previously described [20], [26]. Briefly, for quantitation, sets of cells were cultured and stained simultaneously, and imaged using identical settings. The protein signals were analysed after thresholds were set, such that recognizable clusters were included in the analysis. After subtracting the background, the number, area and the integrated intensity of hnRNP A2/B1 particles in dendrites was determined, and represented per dendritic area (defined by MAP2 staining area). For colocalization analysis, regions around thresholded puncta were overlaid as a mask in the PSD95 channel, and the integrated intensity, area and number of colocalized particles determined. All analyses were done as blind to the experimental condition. For the analysis of hnRNP A2/B1 protein in the neuronal soma, the intensity of hnRNP A2/B1 staining was measured in similar regions of interest in the cell body, outside of the nucleus, using ImageJ image analysis software.

### Preparation of synaptoneurosomes and sucrose gradients

Synaptoneurosomes were prepared from adult rats (12–13 weeks) as described previously [27], with minor alterations. Synaptoneurosomes were lysed in 900 µl of lysis buffer [15 mM Tris-HCl pH 8, 5 mM MgCl_2_, 0.3 M NaCl, 0.5 mM DTT, 0.1 mg/ml Cycloheximide and 1% Triton X-100] supplemented with a cocktail of protease inhibitors (0.1 mM PMSF; CLAP: 1 µg/ml chymostatin, 1 µg/ml leupeptin, 1 µg/ml antipain, 1 µg/ml pepstatin; Sigma-Aldrich) and 100 U/ml of RNase inhibitor (SUPERaseIn, Ambion Applied Biosystems). Membranous structures were removed by spinning at 12,000× g for 10 min. The resulting supernatant was loaded on a 10–50% linear sucrose gradient [prepared in 20 mM Tris-HCl pH 8, 140 mM KCl, 5 mM MgCl_2_, 0.5 mM DTT, 0.1 mg/ml Cycloheximide and 10 U/ml of RNase inhibitor (SUPERaseIn, Ambion Applied Biosystems)] and spun at 35,000 rpm for 190 min (4°C) using a SW41 rotor (Beckman Coulter). Each gradient was separated into 11 fractions, with approximately 1.0 ml each.

### Western blotting

Equal volumes (30 µl) of each fraction isolated from the 10–50% linear sucrose gradient were denatured and protein samples were separated by SDS-PAGE, transferred to PVDF membranes (Millipore), and immunoblotted as described previously [23]. The following primary antibodies were used: anti-hnRNP A2/B1 (sc-53531, 1∶500; Santa Cruz Biotechnology), anti-Staufen1 (AB5781, 1∶500; Millipore), anti-eEF2 (ab40812, 1∶12.500; Abcam) and anti-rpS6 (2217, 1∶1000; Cell Signaling Technology).

### RNA extraction, cDNA synthesis and quantitative RT-PCR

Total RNA was extracted from hippocampal neurons cultured in 3 µm pore 24 mm PET membrane filter inserts using TRIzol Reagent (Invitrogen) as described previously [24]. RNA quality and integrity was assessed using the Experion automated gel-electrophoresis system (Bio-Rad) and the RNA concentration determined using NanoDrop (Thermo Scientific).

For mRNA measurements, 500–1000 ng of total RNA was reverse transcribed using the iScript cDNA Synthesis Kit (170–8891; Bio-Rad) following manufacturer's guidelines. Quantitative RT-PCR was performed using SsoFast EvaGreen Supermix (172–5201; Bio-Rad) and the iQ5 Multicolor Real-Time PCR Detection System (Bio-Rad). 2 µl of 1∶10 diluted cDNA was used and the final concentration of each primer was 250 nM. Rat *Ppia* (Peptidylprolyl isomerase A) was chosen as normalization control since it shows a stable expression in hippocampal neurons stimulated with BDNF [24], [28]. Primers for qRT-PCR are listed as follows: hnRNP A2 forward: 5′-GCTACGGAGGTGGTTATG-3′, reverse: 5′-AGTTAGAAGGTTGCTGGTTAT-3′; *Ppia* forward: 5′-TTTGGGAAGGTGAAAGAAGGC-3′, reverse: 5′-ACAGAAGGAATGGTTTGATGGG-3′. The comparative Ct method was used to quantitate the relative gene expression across the experimental conditions. Data analysis of the log-transformed expression data was performed using GenEx (MultiD Analysis) software for Real-time PCR expression profiling.

### Statistical analysis

Statistical analysis was performed using one way ANOVA followed by the Dunnett's or Bonferroni's test as indicated in the figure captions.

## Results

**Figure 1:**
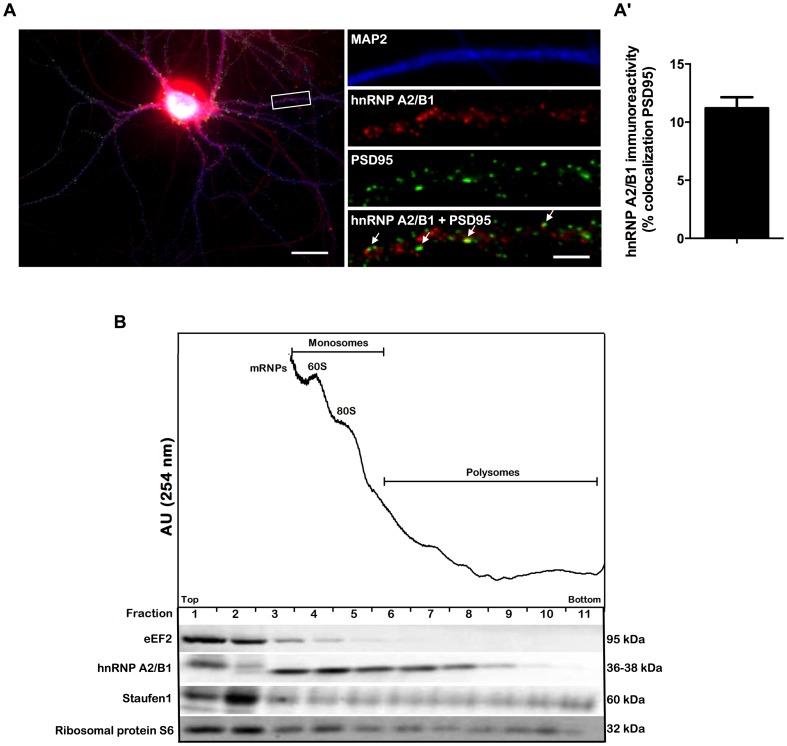
hnRNP A2/B1 is present in synaptic polyribosomal fractions and colocalizes with PSD95 in dendrites of hippocampal neurons. (A) Cultured hippocampal neurons immunostained for MAP2 (blue), hnRNP A2/B1 (red) and PSD95 (green) show that hnRNP A2/B1 is present in synaptic sites as indicated by the colocalization with the postsynaptic marker PSD95 (white arrows). Scale bars = 25 µm and 4 µm for low and high magnification images, respectively. The images are representative of six different experiments performed in independent preparations, with a total of 71 cells analysed. (A′) The percentage of dendritic hnRNP A2/B1 signal that colocalizes with PSD95 was analysed using ImageJ software (mean ± SEM.). (B) Co-sedimentation of synaptoneurosomal proteins using a 10–50% linear sucrose gradient. The polyribosomes, monosomes and mRNPs (non-polysomal fractions) were detected by UV absorbance at 254 nm and the gradient was collected in 11 fractions. Equal volumes from each fraction were analysed by SDS-PAGE and Western blot using antibodies that recognize hnRNP A2/B1, Staufen1, eEF2 and ribosomal protein S6. The results are representative of two different experiments performed in independent synaptoneurosomal preparations.

hnRNP A2/B1 is required for the dendritic localization of mRNAs encoding proteins that are relevant for synaptic plasticity, such as CaMKIIα and Arc [7]. Dendritic-localized mRNAs are typically transported in a dormant state until the translational block is relieved upon activity at synaptic domains [4]. To test if hnRNP A2/B1 is present at the synapse, we analysed the colocalization with the postsynaptic marker PSD95, in cultured hippocampal neurons (Fig. 1A; 1A′). A considerable fraction of dendritic hnRNP A2/B1 (11.2±0.9%) localizes at PSD95-positive clusters (Fig. 1A-white arrows; 1A′). Similarly, a significant percentage of total PSD95-positive synapses contain hnRNP A2/B1 (7.7±0.5%; n = 48 cells) (data not shown). In addition, we detected hnRNP A2/B1 and Staufen1, another RNA-binding protein present in neuronal RNA granules [29], [30], in free mRNPs-, monosomal-, and polyribosomal- associated fractions obtained from rat hippocampal synaptoneurosomes (Fig. 1B). hnRNP A2/B1 was particularly enriched in monosome fractions (Fig. 1B). Altogether, these findings suggest that hnRNP A2/B1 can localize in synaptic domains under resting conditions.

**Figure 2:**
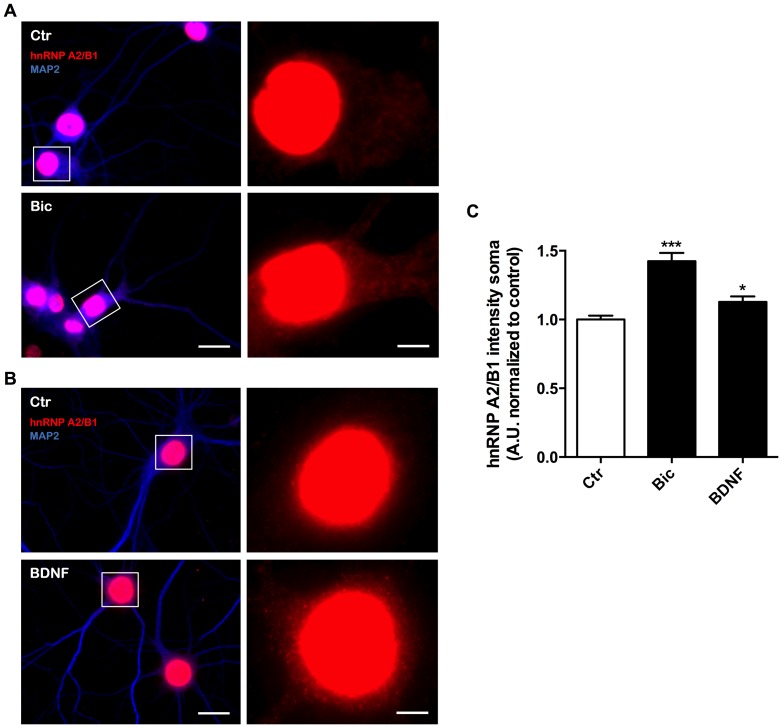
Synaptic activity and the neurotrophin BDNF increase the abundance of hnRNP A2/B1 protein in the soma of hippocampal neurons, outside the nucleus. (A) Cultured hippocampal neurons were stimulated or not with bicuculline (50 µM), 4-AP (2.5 mM) and glycine (10 µM) for 30 min and then the cells immunostained for MAP2 (blue), hnRNP A2/B1 (red). Scale bar = 20 µm and 4 µm for low and high magnification images, respectively. (B) Cultured hippocampal neurons were stimulated or not with BDNF (100 µg/ml), for 30 min and then the cells immunostained for MAP2 (blue), hnRNP A2/B1 (red). Scale bar = 20 µm and 4 µm for low and high magnification images, respectively (C) The intensity of hnRNP A2/B1 protein in the cell body was analysed in similar regions of interest within the soma, away from the nucleus, using ImageJ software. Results are normalized to control and are averaged of 4–7 different experiments performed in independent preparations. The following number of cells was used in the analysis of the somatic-localized hnRNP A2/B1 Ctr (n = 71 cells), Bic (n = 40 cells), BDNF (n = 61 cells). Error bars, mean ± SEM. Statistical analysis was performed by one-way ANOVA, followed by Dunnet's test.* P<0.05; *** P<0.001.

**Figure 3:**
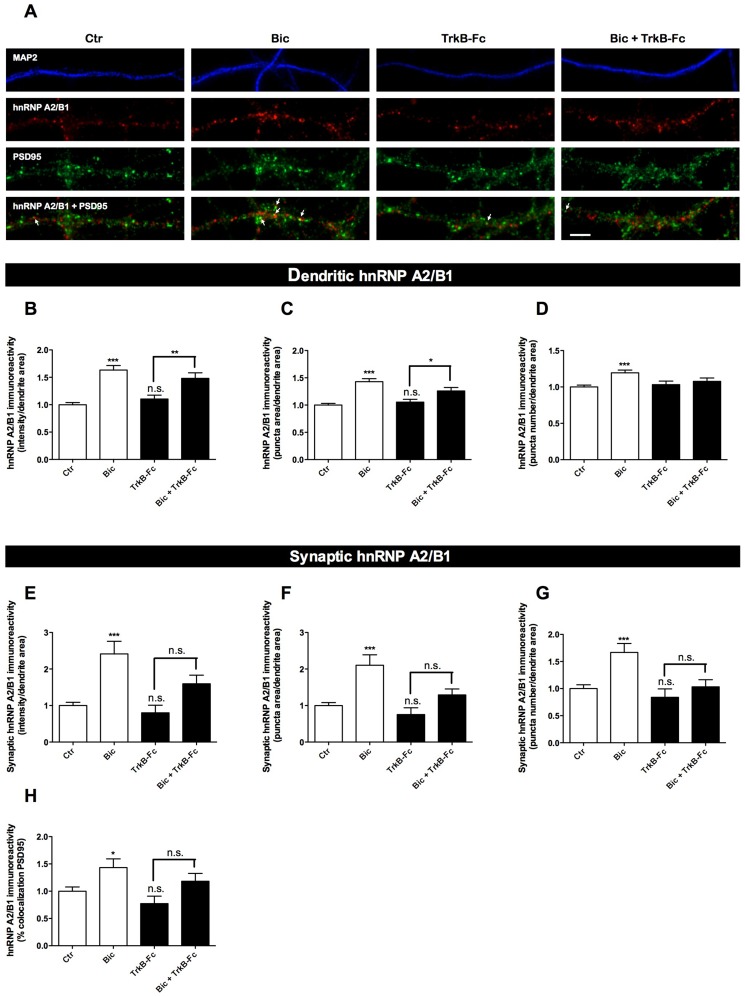
Synaptic activity increases hnRNP A2/B1 in dendrites and induces the delivery of hnRNP A2/B1 to the synapse by a BDNF-dependent mechanism. Cultured hippocampal neurons were stimulated or not with bicuculline (50 µM), 4-AP (2.5 mM) and glycine (10 µM), for 30 min. Where indicated, neurons were treated with TrkB-Fc (1 µg/ml) for 30 min and then stimulated or not with bicuculline in the presence of the BDNF scavenger. The cells were immunostained for hnRNP A2/B1 (red), MAP2 (blue), and PSD95 (green) (A). White arrows indicate PSD95-positive clusters that also contain hnRNP A2/B1 (A). The integrated fluorescence intensity, area and number of hnRNP A2/B1 puncta in dendrites (B, C, and D) and at the synapse (as defined by the signal of hnRNP A2/B1 that overlaps with PSD95) (E, F, and G) was analysed using ImageJ software and represented per dendritic area. The percentage of dendritic hnRNP A2/B1 signal that colocalizes with PSD95 was also analysed (H). Results are normalized to control and are averaged of 3–7 different experiments performed in independent preparations. The following number of cells was used in the analysis of the dendritic-localized hnRNP A2/B1 puncta: Ctr (n = 87 cells), Bic (n = 64 cells), TrkB-Fc (n = 47 cells), TrkB-Fc+Bic (n = 48 cells). For the analysis of synaptic-localized hnRNP A2/B1 puncta the following number of cells was analysed: Ctr (n = 76 cells), Bic (n = 54 cells), TrkB-Fc (n = 35 cells), TrkB-Fc+Bic (n = 35 cells). Error bars, mean ± SEM. Statistical analysis was performed by one-way ANOVA, followed by Bonferroni's test. n.s. Not significant, * P<0.05; ** P<0.01; *** P<0.001. Scale bar = 4 µm.

Several transcripts are transported to dendrites and dendritic spines upon neuronal activation, including the mRNAs for Arc [10]–[13], β-actin [31], CaMKIIα [14]–[16], TrkB and BDNF [17]. The mRNAs encoding for Arc, CaMKIIα and BDNF have A2RE sequences in their sequence [7], [18], and the Arc and CaMKIIα transcripts appear to be transported along dendrites in an hnRNP A2/B1-dependent manner [7]. However, it remains to be determined whether the hnRNP A2/B1-dependent mRNA transport is a constitutive or regulated process. Therefore, we investigated whether synaptic activity changes the cytoplasmic levels of hnRNP A2/B1 and regulates the delivery of this protein into synapses. For that purpose, cultured hippocampal neurons were stimulated for 30 min with a cocktail solution with bicuculline to increase the excitatory activity of the neuronal network [32]. Bicuculline treatment significantly increased the intensity of hnRNP A2/B1 in the soma compartment (Fig. 2A,C). Similarly, synaptic activity increased the integrated intensity, as well as the number and area of hnRNP A2/B1 puncta in dendrites (Fig. 3A–D) and in synaptic sites (Fig. 3A-white arrows, E–G). Since the increase in synaptic levels of hnRNP A2/B1 could be a result of the general increase observed in dendrites, we also analysed the percentage of dendritic hnRNP A2/B1 signal that overlaps with the synaptic marker PSD95. Synaptic activity also increased the percentage of dendritic hnRNP A2/B1 that colocalizes with PSD95 (Fig. 3H), suggesting that there is a preferential increase in the delivery of hnRNP A2/B1 into synaptic sites, which is not only due to the overall change that occurs in dendrites.

The neurotrophin BDNF plays a key role in several forms of synaptic plasticity [33]. Some of the actions of BDNF in the CNS rely, in part, on the ability of this neurotrophin to change the synaptic proteome through the regulation of the delivery of dendritic-localized transcripts and by regulating local protein synthesis at the synapse [3], [34]. Several activity-inducing paradigms were shown to promote the release of endogenous BDNF [35], [36]. Thus, we investigated whether the endogenous released BDNF was involved in the bicuculline-induced regulation of hnRNP A2/B1. For that purpose we used the TrkB-Fc chimera, an effective scavenger of TrkB ligands which has been widely used to access the endogenous functions of BDNF in the CNS. TrkB-Fc did not prevent bicuculline-induced increase in dendritic hnRNP A2/B1 puncta intensity and area (Fig. 3A–C) but partially prevented the increase on synaptic hnRNP A2/B1 observed upon bicuculline treatment (Fig. 3A, E–G). Further analysis of the data demonstrated that bicuculline-induced increase in hnRNP A2/B1 puncta number at the synapse is significantly reduced (P<0.05) in the presence of the scavenger TrkB-Fc, as determined by comparing the Δ between Bic/Ctr and Bic+TrkB-Fc/TrkB-Fc experimental conditions (data not shown). A similar approach allowed showing that the bicuculline-induced increase in hnRNP A2/B1 in dendrites is of less magnitude when TrkB-Fc is present (data not shown).

Altogether, our data indicate that the activity-dependent delivery of hnRNP A2/B1 into synaptic sites likely depends on the release of BDNF and extracellular activation of TrkB receptors. In contrast, the accumulation of hnRNP A2/B1 in dendrites upon synaptic activity still occurred when the extracellular BDNF was quelated suggesting that it may have a component that does not require the actions of endogenous released BDNF. However, the magnitude of the abovementioned increase seems to be affected by the presence of the scavenger (data not shown) thus, one cannot exclude the possibility of BDNF-TrkB signaling being involved, in part, in the activity-dependent accumulation of hnRNP A2/B1 in dendrites.

**Figure 4:**
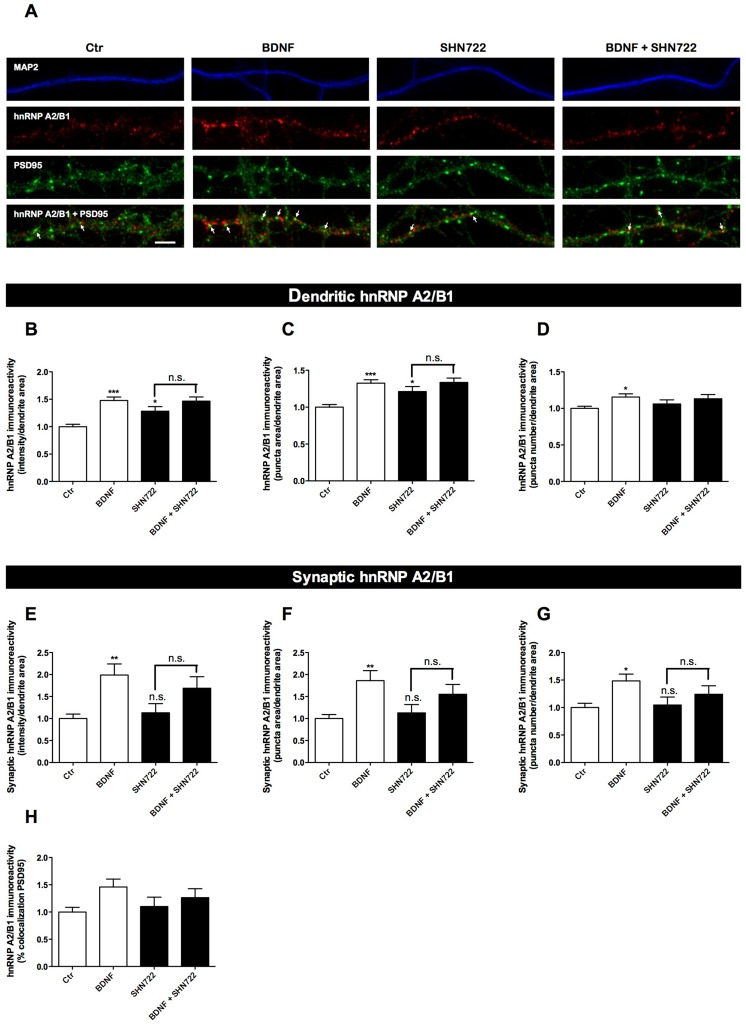
BDNF induces the accumulation of hnRNP A2/B1 in dendrites and at the synapse in hippocampal neurons. Cultured hippocampal neurons were stimulated or not with 100 ng/ml BDNF for 30 min. The role of Trk receptor activity in BDNF-induced regulation of hnRNPA2/B1 distribution was tested using the inhibitor SHN722. Where indicated, neurons were treated 30 min with 1 µM SHN722 and then stimulated or not with 100 ng/ml BDNF in the presence of the inhibitor. The cells were immunostained for hnRNPA2/B1 (red), MAP2 (blue), and PSD95 (green) (A). White arrows indicate PSD95-positive clusters that also contain hnRNP A2/B1 (A). The integrated fluorescence intensity, area and number of hnRNP A2/B1 puncta in dendrites (B, C, and D) and at the synapse (as defined by the signal of hnRNP A2/B1 that overlaps with PSD95) (E, F, and G) was analysed using ImageJ software and represented per dendritic area. The percentage of dendritic hnRNP A2/B1 signal that colocalizes with PSD95 was also analysed (H). Results are normalized to control and are the average of 3–6 different experiments performed in independent preparations. The following number of cells was used in the analysis of the dendritic-localized hnRNP A2/B1 puncta: Ctr (n = 73 cells), Bic (n = 64 cells), TrkB-Fc (n = 47 cells), TrkB-Fc+Bic (n = 48 cells). For the analysis of synaptic-localized hnRNP A2/B1 puncta, the following number of cells was analysed: Ctr (n = 62 cells), Bic (n = 67 cells), TrkB-Fc (n = 37 cells), TrkB-Fc+Bic (n = 35 cells). Error bars, mean ± SEM. Statistical analysis was performed by one-way ANOVA, followed by Bonferroni's test. n.s. Not significant, * P<0.05; ** P<0.01; *** P<0.001. Scale bar = 4 µm.

We next questioned whether exogenous BDNF alters the cytoplasmic distribution of hnRNP A2/B1 in hippocampal neurons. Stimulation of cultured hippocampal neurons with BDNF (100 ng/ml) for 30 min resulted in a significant increase in hnRNP A2/B1 levels in the neuronal soma (Fig. 2B,C). To investigate if BDNF alters the dendritic and synaptic distribution of hnRNP A2/B1 cultured hippocampal neurons were stimulated with BDNF (100 ng/ml) for 30 min, in the presence or in the absence of the Trk receptor inhibitor SHN722 (1 µM) [21], [22]. BDNF treatment resulted in a significant increase of hnRNP A2/B1 integrated intensity, area and puncta number in dendrites (Fig. 4A–D) and in synaptic sites (Fig. 4A-white arrows, E–G). Inhibition of Trk receptor activity with SHN722 induced a modest increase in hnRNP A2/B1 puncta area and integrated intensity in dendrites (Fig. 4A–C). Nevertheless, further stimulation with BDNF had no significant effect in every parameter evaluated in both dendritic and synaptic hnRNP A2/B1 particles, showing that the effects of BDNF were mediated by Trk receptors. Accordingly, further analysis of the data allowed demonstrating that the BDNF-induced increase in dendritic and synaptic hnRNP A2/B1 is reduced, significantly in the former case, in the presence of the inhibitor SHN722 (comparing the Δ between BDNF/Ctr and BDNF+SHN722/SHN722 experimental conditions) (data not shown). Taken together, our findings indicate that BDNF increases the levels of hnRNP A2/B1 in the cell body and induces a robust accumulation of hnRNP A2/B1 in dendrites and at the synapse in hippocampal neurons. The latter effects are mediated by the activation of Trk (presumably TrkB) receptors.

**Figure 5:**
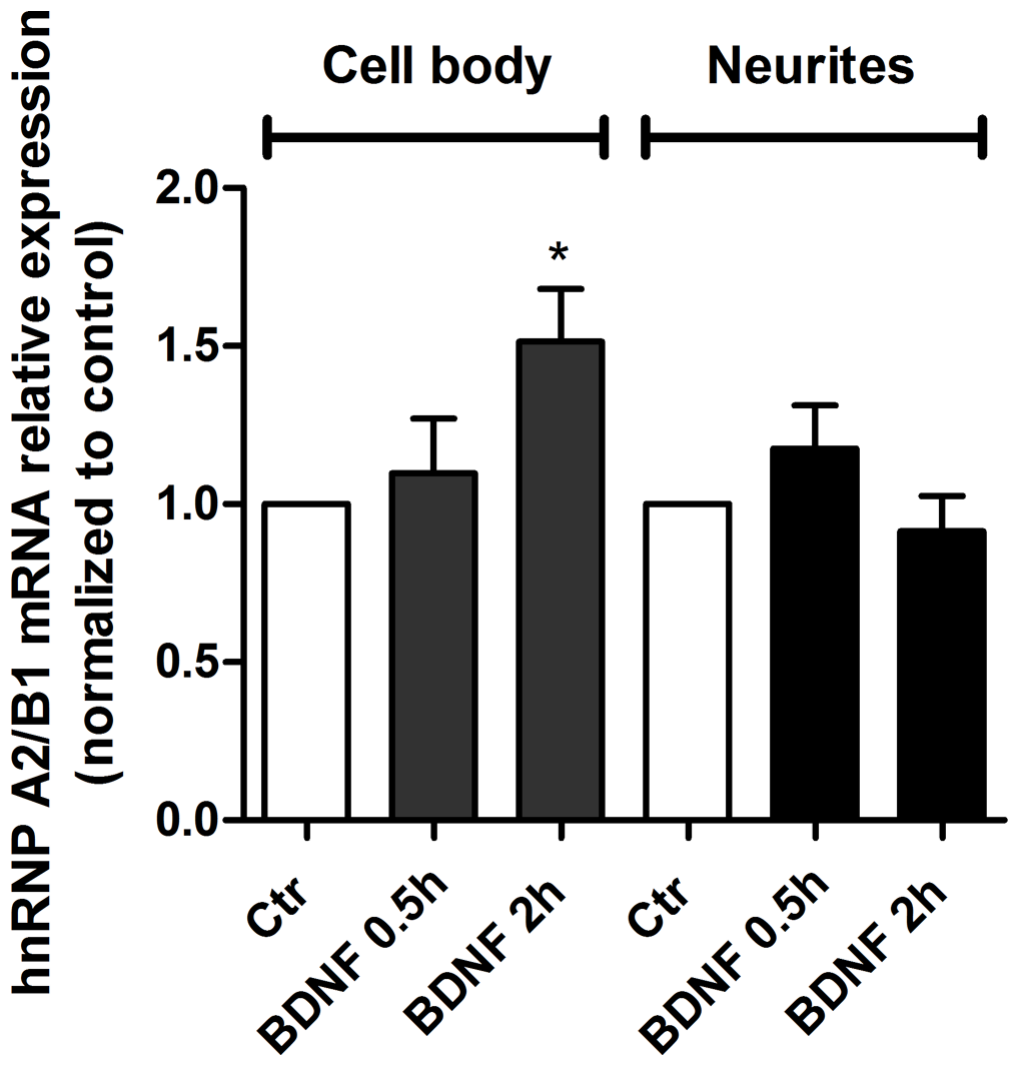
BDNF upregulates hnRNP A2/B1 mRNA in the cell body compartment of hippocampal neurons. Cultured hippocampal neurons were stimulated or not with 100 ng/ml BDNF, for 30 min or 2 h. The cell body mRNA was mechanically separated from the transcripts of neurites and 500–1000 ng of RNA from each compartment was used in the reverse transcription reaction. The analysis of hnRNP A2/B1 mRNA levels was performed by qRT-PCR using *Ppia* as internal control gene. The results are the average ± SEM of five (cell body compartment) or seven (neurite compartment) independent transcription reactions, performed in distinct preparations. Statistical analysis of logtransformed expression data was performed by one-way ANOVA, followed by Dunnet's test. * P<0.05.

The dendritic transcriptome is not yet fully characterized but a recent study using deep RNA sequencing in microdissected synaptic neuropil (*stratum radiatum* and *lacunosum moleculare*) segments from the CA1 region of the adult rat hippocampus allowed the identification of approximately 2550 mRNAs in dendrites and/or axons [37], including the hnRNP A2/B1 mRNA [37]. Using a neuronal culture system that allows the mechanical separation of neurites from cell bodies [24], [25] we investigated the effect of BDNF on the levels of hnRNP A2/B1 transcripts in the two compartments. qRT-PCR experiments showed that BDNF treatment for 2 h significantly increased the hnRNP A2/B1 mRNA in the soma but had no effect on the abundance of the transcript in the neurite compartment (Fig. 5).

## Discussion

In this work we show that hnRNP A2/B1 is commonly detected in dendrites and in synaptic sites of cultured hippocampal neurons under resting conditions. Furthermore, we found that hnRNP A2/B1 protein levels are rapidly increased in the cell body, dendrites and synapses of hippocampal neurons upon neuronal activation or BDNF stimulation. Together, these results point to a tight regulation of the cytoplasmic distribution of hnRNP A2/B1, and do not favor a model of constitutive delivery of the protein into dendrites. This is particularly relevant since hnRNP A2/B1 is a *trans-*acting factor involved in the transport of several mRNAs along dendrites [7]–[9], [38].

3BDNF plays an important role in the protein synthesis-dependent late phase of LTP induced by high-frequency stimulation in the hippocampus CA1 region [39], [40]. BDNF-induced synaptic potentiation has also been reported, both *in vitro* [41] and *in vivo* [42], [43]. The observed increase in the dendritic distribution and synaptic clustering of hnRNP A2/B1 in hippocampal neurons stimulated with BDNF suggest that the neurotrophin may act, at least in part, by regulating the transport of mRNAs during plasticity-related events. In particular, the BDNF-induced clustering of hnRNP A2/B1 at the synapse may bring the Arc and CaMKII mRNAs (among others) that are locally translated and may contribute to the protein synthesis-dependent late phase of LTP. Accordingly, intrahippocampal infusion of BDNF resulted in the accumulation of Arc transcripts in dendrites and triggered long-term potentiation (BDNF-LTP) at medial perforant path-granule cell synapses *in vivo* [43], [44]. Furthermore, exogenous application of BDNF is sufficient to induce the transport of A2RE-containing mRNAs, such as BDNF [45] and Arc [46] transcripts, into dendrites.

Although synaptic activity and BDNF stimulation increased hnRNP A2/B1 protein levels and clustering in dendrites, the effect of neuronal activity was in part insensitive to the presence of TrkB-Fc, indicating that it might have a component that is not mediated by the release of endogenous BDNF. This contrasts with the synaptic delivery of the protein upon neuronal activation, which is likely to require the activation of TrkB receptors by BDNF. Further research is needed to clarify the differential mechanisms induced by synaptic activity and BDNF that promote the accumulation of hnRNP A2/B1 in dendrites.

It was shown that BDNF treatment increases the proportion of motile DEAD box 3 helicase-carrying RNA granules in dendrites of hippocampal neurons [47]. Since these granules are believed to contain hnRNP A2/B1, one may speculate that the increased motility of RNA granules in response to BDNF might contribute to the BDNF-induced synaptic accumulation of hnRNP A2/B1. At the synapse hnRNP A2/B1 may release the transcripts that specifically bind to this ribonucleoprotein in response to specific stimuli, such as stimulation of TrkB receptors by BDNF. Accordingly, phosphorylation of hnRNP A2/B1 by the Fyn kinase was correlated with the increased translation of a MBP mRNA reporter in oligodendrocytes [48] and TrkB receptors were shown to activate Fyn in the hippocampus [49].

Among the pleiotropic roles of BDNF in the mammalian brain, is the capacity of the neurotrophin to induce the formation of new synapses [50]. It was recently demonstrated that hippocampal synaptogenesis requires the BDNF-mediated regulation of the motor protein KIF1A and KIF1A-mediated cargo transport [51]. However, it remains to be determined whether KIF5, which is responsible for the transport of RNA containing granules [52], or myosin-Va, which facilitates the transport of mRNP complexes to dendritic spines [53], are regulated by BDNF-induced signaling.

The fractionation studies showed that hnRNP A2/B1 is present in both monosomal- and polyribosomal-associated fractions at the synapse. Because polyribosomes are sites of active translation [54], [55], and since the translation of dendritic-localized mRNAs is also thought to occur via monosomes [56], these results suggest that, besides its role in mRNA transport, hnRNP A2/B1 may also regulate the translation of the cognate mRNAs at the synapse. In agreement with these findings, hnRNP A2/B1 was shown to be player in the regulation of localized translation of MBP mRNA in oligodendrocytes [57]. Since dendritic mRNAs are typically transported in a translational silent state, it will be important to investigate whether hnRNP A2/B1 has the ability to repress the translation of the target mRNAs during transport in neurons.

In the present study we also provide evidence for the presence of Staufen1 in synaptic polysomal fractions (Fig. 1B). Staufen1 is another well described *trans-*acting factor localized in dendritic RNA granules [29], [30] that regulates the transport of mRNA [52], [58] and was shown to play a role in protein synthesis-dependent LTP in hippocampal pyramidal neurons [59]. Our results suggest a role for this protein not only in the delivery but also in the translational control of synaptic-localized mRNAs. To our knowledge this is the first indication that Staufen1 may play a role in the translational control at the synapse in mammalian neurons. This is in agreement with the results showing the presence of Staufen proteins in polysomal fractions isolated from COS7 and HeLa cell lines [60], [61], and with the role of Staufen1 in the translational control of mRNAs [62].

Using a culture system that allows a physical separation of the soma and neurite compartment, we found that stimulation of cultured hippocampal neurons with BDNF increased the hnRNP A2/B1 mRNA only in the former compartment. The BDNF-induced upregulation of hnRNP A2/B1 mRNA in cell bodies may provide a layer to support the effect of the neurotrophin on the hnRNP A2/B1 protein localization in the cytoplasm or even to contribute to the wide variety of functions that hnRNP A2/B1 plays in the nucleus. The lack of effect of BDNF on the dendritic levels of hnRNP A2/B1 mRNA contrasts with the effects observed for transcripts encoding several translation-related proteins [24], and indicates that the transport of the mRNAs for this ribonucleoprotein is also tightly regulated. The differential effect of BDNF on the dendritic levels of hnRNP A2/B1 mRNA and protein may be due to the specificities of the transport of mRNAs vs proteins.

Overall, our study provides strong evidence supporting the dendritic accumulation of hnRNP A2/B1 in response to synaptic activity and upon BDNF treatment, most likely through distinct mechanisms. Similarly, the activity-dependent BDNF-mediated synaptic delivery of hnRNP A2/B1 further suggests a role for hnRNP A2/B1 in local mRNA metabolism and is likely to have a role in BDNF-mediated synaptic plasticity events.
